# Supplementary material for: Chromothripsis during telomere crisis is independent of NHEJ, and consistent with a replicative origin
Source: Genome Res. 2019 May;29(5):737–49. doi: 10.1101/gr.240705.118 (PMC6499312; doi:10.1101/gr.240705.118)
Supplement: Supplemental Material [file supp_gr.240705.118_Supplemental_file_1.zip › contigs/annotated_contigs/DB53/contig.2.DB53_length_652_mean_cov_6.4754601227.docx]

**DB53_length_652_mean_cov_6.4754601227**

```
TACTTGGGAGGCTGAGGCAGGAGAATCACCTGAACCCAGGAGGCAGAGGCTGCAGTGAGCTGAGATTGTGCCACCGCACTCCAGCCTGG
 >chr3:69971169-69971502 - E=1e-188 p=0e+00
CGACAGAGTGAGACTCCGTCTGAAAAAAAAAGCAATGGCATAGTCAATCCTCAAATCTCTATATATTGTGTGGTAGACAGAAAAGAAAG

CGAGACCTCAACATGAAAGAAAATACTTCATTTCTATCATCAAGGGGCTTACAACCCAAGGGTTAGGTGGGGGAAACAGGGAGAGTAAC

CCACACAGAAATTTACAGTAAGGTGCTCCTGCCTATTAAGATGGGAGCTCTCAACTGTAC|AGGCAG|GGGAATTGCTTGAACCAGGGA
                                                             >chr3:69969809-69970134 -
GGTGGAGGTTGCAGTGAGCCGAGGTCGAGCCACTGCACTCCAGCCTGGGCAACAGAGTGAGACTCTGTCTTGTAGGGACCAGCCCCACA
E=8e-184
GGGTCGGTGGGTCTCTCCCCGTGTGCAGTGACGAGAGAGTGTAGAAATAAAGACACAAGACAAAGAGATAAAAGAAAAGACAGCTGGGC

CCGGGGAACCACTACCACAAATGCGCGGAGACCGGTAGTGGCCCCGAATGTCTGGCTGCACTGTTATTTATTGGATACAAAGCAAAAGG

GGCAGGGTAAAGAGTGTGAGTCATCTCCAAT
```
